# Evolution of unexpected diversity in a putative mating type locus and its correlation with genome variability reveals likely asexuality in the model mycorrhizal fungus *Rhizophagus irregularis*

**DOI:** 10.1186/s12864-024-10770-9

**Published:** 2024-09-20

**Authors:** Soon-Jae Lee, Eric Risse, Ivan D. Mateus, Ian R. Sanders

**Affiliations:** Department of Ecology and Evolution, University of Lausanne, Lausanne, 1015 Switzerland

**Keywords:** Arbuscular mycorrhizal fungi (AMF), *Rhizophagus irregularis*, Mating-type, Recombination, Asexuality, Asexual reproduction, Loss of sex

## Abstract

**Background:**

Arbuscular mycorrhizal fungi (AMF) form mutualistic partnerships with approximately 80% of plant species. AMF, and their diversity, play a fundamental role in plant growth, driving plant diversity, and global carbon cycles. Knowing whether AMF are sexual or asexual has fundamental consequences for how they can be used in agricultural applications. Evidence for and against sexuality in the model AMF, *Rhizophagus irregularis,* has been proposed. The discovery of a putative mating-type locus (MAT locus) in *R. irregularis,* and the previously suggested recombination among nuclei of a dikaryon *R. irregularis* isolate, potentially suggested sexuality. Unless undergoing frequent sexual reproduction, evolution of MAT-locus diversity is expected to be very low. Additionally, in sexual species, MAT-locus evolution is decoupled from the evolution of arbitrary genome-wide loci.

**Results:**

We studied MAT-locus diversity of *R. irregulari*s. This was then compared to diversification in a phosphate transporter gene (PTG), that is not involved in sex, and to genome-wide divergence, defined by 47,378 single nucleotide polymorphisms. Strikingly, we found unexpectedly high MAT-locus diversity indicating that either it is not involved in sex, or that AMF are highly active in sex. However, a strongly congruent evolutionary history of the MAT-locus, PTG and genome-wide arbitrary loci allows us to reject both the hypothesis that the MAT-locus is involved in mating and that the *R. irregularis* lineage is sexual.

**Conclusion:**

Our finding shapes the approach to developing more effective AMF strains and is highly informative as it suggests that introduced strains applied in agriculture will not exchange DNA with native populations.

**Supplementary Information:**

The online version contains supplementary material available at 10.1186/s12864-024-10770-9.

## Background

The symbiosis between plants and arbuscular mycorrhizal fungi (AMF; phylum Glomeromycota) is one of the most successful mutualistic partnerships on earth. The fungi colonize the roots of vascular plants and help plants acquire various nutrients (especially phosphate) from the soil, as well as mitigating plant stress [1]. The symbiosis occurs with plants in almost all terrestrial ecosystems [2], influencing global ecosystem functioning, especially carbon, phosphate and nitrogen cycles. Genetic variation in AMF differently alters plant productivity and growth, [3–8]. Identifying the mechanisms influencing or maintaining the genetic diversity in these important fungi is, thus, crucial for understanding global ecosystem functioning.

Even though AMF have successfully lived with plants in terrestrial ecosystems for approximately 460 mya [1], how the genetic diversity is generated and maintained in AMF remains largely unknown. Mating and consequent sexual recombination are considered to be the fundamental mechanisms for generating genetic diversity in Eukaryotes. Previously, AMF were considered as ancient asexuals [9]. This was based on circumstantial evidence; namely, the lack of any observed sexual structures and the finding of AMF-like structures in fossilized roots of the earliest land plants [1]. These fungi are unique in that they form multi-nucleated spores (several hundreds to thousands nuclei per single spore) and never produce a single or two nucleus stage in their whole lifecycle [1] This highlights the a physiological difficulty for mating and sexual recombination in AMF. More recently, molecular studies have shown that AMF nuclei are haploid and to date no diploid nuclei have ever been observed [1, 10]. More recently, evidence for sexuality in AMF has been suggested. Three lines of evidence support possible sexual reproduction in AMF. First, the genome of the model AMF species, *Rhizophagus irregularis*, contains a conserved partial set of genes thought necessary for meiosis, although the role of these genes in AMF has not been demonstrated [11]. Second, putative recombination in an AMF population has been suggested [12, 13]. Third, *R. irregularis* exists as homokaryons (carrying a population of genetically identical haploid nuclei) and as dikaryons (carrying a population of two different haploid nucleus genotypes) even though the stage is not diploid. Although AMF lack most of the known sex loci involved in fungal mating, a genomic region was identified in *R. irregularis* that is similar to a mating type locus (MAT-locus) of Basidiomycetes; a fungal phylum that is evolutionarily distant from the Glomeromycota [14]. In *R. irregularis,* each haploid nucleus carries one copy of the MAT-locus [14]. To date, all *R. irregularis* dikaryons have been shown to possess two different MAT alleles [14]. MAT-loci define the sexual identity of fungi in all uni- or bifactorial mating systems found in different fungal lineages [15]. The MAT-locus in Basidiomycetes contains genes encoding homeodomain transcription factors named HD1 and HD2. Allelic variation of these genes determines sexual compatibility with other individuals. In heterothallic fungi, only individuals with different alleles at the MAT-locus can engage in sexual reproduction [16–18]. For an organism with facultative or rare sex, the diversity of MAT-types is expected to remain low due to the difficulties for rare MAT-type maintenance. Indeed, most sexual fungi have only two MAT-types [19]. There are some extreme cases in obligatory sexual fungi where high numbers of MAT-types have evolved and maintained. For example, *Coprinellus disseminatus* has 143 MAT-types and *Schizophyllum commune* has > 23,000 MAT-types [15, 20] and *Trichaptum* species have over 17,000 predicted MAT-types across 2 MAT-loci [21]. However, in these fungi, frequent mating is necessary to ensure that rare MAT-alleles will not go to extinction [19].

In other fungi, MAT-locus can be also involved in functions that are not related to sexual reproduction, such as asexual sporulation and related cell cycle regulation [22]. Therefore, the existence of the MAT-locus does not prove sexuality in this organism. Nevertheless, the existence of this MAT-locus in AMF has become a focus candidate indicating sex because of the lack of any other promising candidate loci. The current sex model in AMF assumes MAT-locus based non-self-recognition, formation of diploid nuclei followed by eventual sexual recombination to generate genetic diversity [14]. The existence of the locus, with two different alleles located on the two nucleus genotypes in dikaryons formed the basis for recent genomic studies proposing sexual recombination in AMF. Several studies attempted to demonstrate recombination events between nuclei of *R. irregularis* dikaryons carrying different MAT alleles. Chen, et al. [23] surveyed three genetically different dikaryon isolates and found a very small number of potential recombination sites in only one isolate. However, this was questioned by Auxier and Bazzicalupo [24] who suggested that these may be artefacts. A re-analysis of the data from Chen, et al. [21] also revealed a small number of potential recombination sites between nuclei [25]. Sperschneider, et al. [26] conducted similar analyses with phased genome assemblies of dikaryon isolates but could not detect reciprocal recombination between co-existing nuclei. To date, none of those studies were able to determine whether any possible recombination events were from meiotic or mitotic recombination.

Population genomics studies on *R. irregularis* do not support sexuality in this fungus. Genetically highly similar *R. irregularis* isolates found in very distant geographical locations, and even on different continents, is entirely inconsistent with a species that exhibits frequent sexual recombination [27]. Consequently, in *R. irregularis* high MAT-type diversity is not expected. To date, sequencing of the MAT-locus has revealed some diversity with 7 MAT-types among 114 different isolates [14, 28]. However, the clustering of MAT-types reported in previous studies were based on nucleotide sequence similarity, with an arbitrary similarity threshold. Consequently, it is possible that two alleles with similar, but small sequence differences, could be reported as identical MAT-types, even if those sequence differences are non-synonymous. If the MAT-locus confers mating identity, then sequence divergence at the MAT-locus should be carefully investigated in both nucleotide and amino acid sequence levels to define the MAT-types. Consequently, the full diversity of the MAT-locus may not have been elucidated.

Understanding whether AMF are sexual or not is not only essential for understanding their ecology and how they have evolved, but also for their use in agriculture. Enormous variation in the effects of genetically different *R. irregularis* isolates on growth and yield of globally important crops [5, 8] means that understanding how genetic variation in these fungi is generated is essential. Furthermore, the application of AMF in agriculture raises questions about their impact in local environments [29], with the concern that an introduced AMF inoculum may mate with the local population, thus, altering the genetic composition of the local population, with unknown consequences. Finally, knowing whether AMF are sexual or not will also determine whether using genetic variation in AMF to improve crop growth can rely on fungal breeding approaches or whether programs will have to rely on the existing genetic variation in the absence of recombination.

**Fig. 1:**
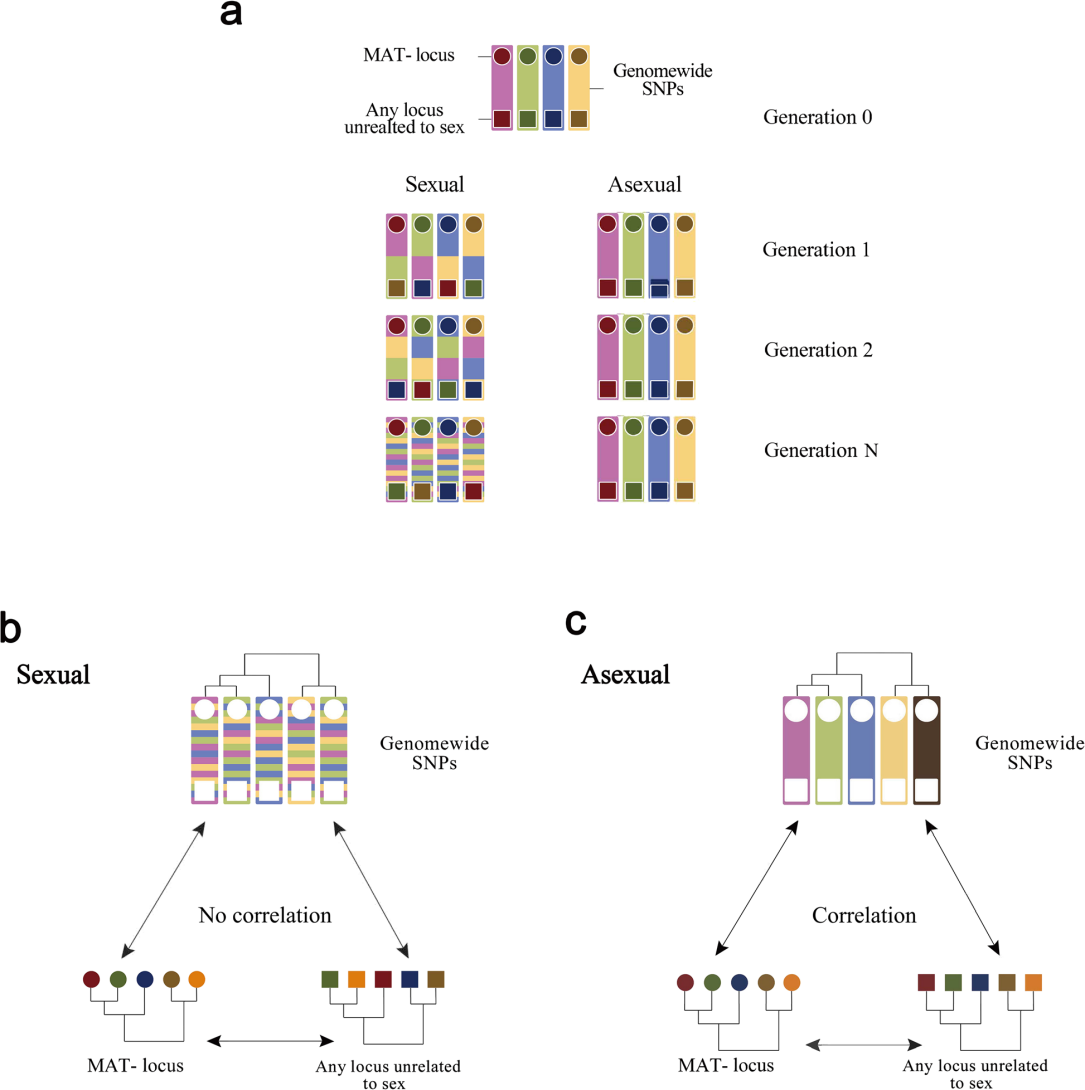
Schematic illustration of hypothetical asexual vs sexual scenarios of genome evolution. **a** Genomes of different individuals represented as coloured bars. The MAT-locus is indicated by a circle while a given arbitrarily chosen locus, that is unrelated to sex, is shown as a square. **b** In the scenario of sexual reproduction, due to genome recombination, there should be no correlation expected among the genome (without MAT-locus and a without the locus containing a gene unrelated to mating or sex) divergence, the MAT-locus divergence and divergence of the gene unrelated to mating or sex. **c** In the scenario of asexuality, the evolution of different loci of the genome, including the MAT-locus (marked as circular shape) and another locus unrelated to mating or sex (in this case, the phosphate transporter gene) will share the same genealogical history because there is no sexual recombination among genomes [30]

Mating and subsequent sexual recombination decouple the evolution of different loci in populations. Loci of asexual organisms share the same genealogical history and, thus, the fate of each locus depends on the fitness afforded by the entire genome [30]. In a population of a sexual species where MAT-locus serves as sex locus, MAT-locus alleles (also sometimes referred to as MAT-types) are expected to display two characteristics: 1) The sequences should be highly conserved, as it determines the mating identity of the organism, and allows non-self recognition which is a prerequisite for mating. 2) The divergence of genes at non-related loci (functionally unrelated, as well as unrelated in genetic distance) or ultimately, the whole genome of a nucleus (represented by thousands of loci) should be independent from MAT-locus identity, because mating decouples the evolution of different loci (Fig. 1) [31–34].

Here, we studied and compared the diversity of the MAT-locus, a phosphate transporter gene (PTG) and genome divergence at arbitrarily chosen loci in the model AMF *R. irregularis* from Africa, Europe and North America in order to address the question regarding sexuality in this important fungal species. The PTG was chosen because functionally it is an important gene in the symbiosis between AMF and plants. Therefore, its divergence reflects functional divergence of the fungi, with respect to the symbiosis. At the same time, the PTG encodes a protein that is not functionally linked to, or expected to be involved in, sex or mating. Genome divergence was based on genome-wide single nucleotide polymorphisms (SNPs) that represents a set of alleles at thousands of arbitrarily chosen loci. We first hypothesized that AMF are sexual and, thus, MAT-type diversity is low compared to genome diversity, as is the case of global yeast populations [32]. In the scenario of sexual reproduction, one gene, or a randomly selected coding region, will not evolve together with all other loci (including both coding and non-coding regions). Therefore, our second hypothesis is that for each pairwise comparison of the 3 data sets (a selected gene which is unrelated to sex or mating, the MAT-locus and genome-wide arbitrary loci), there will be no correlation in between any pair of the 3 datasets. In order to test these hypotheses, we first carried out MAT-locus sequencing of many *R. irregularis* isolates. We then re-analysed all available population datasets available for *Rhizophagus* [27, 35] and newly generated genome and PTG datasets.

## Results

### MAT sequence-based phylogeny discriminates different *Rhizophagus* species

**Fig. 2:**
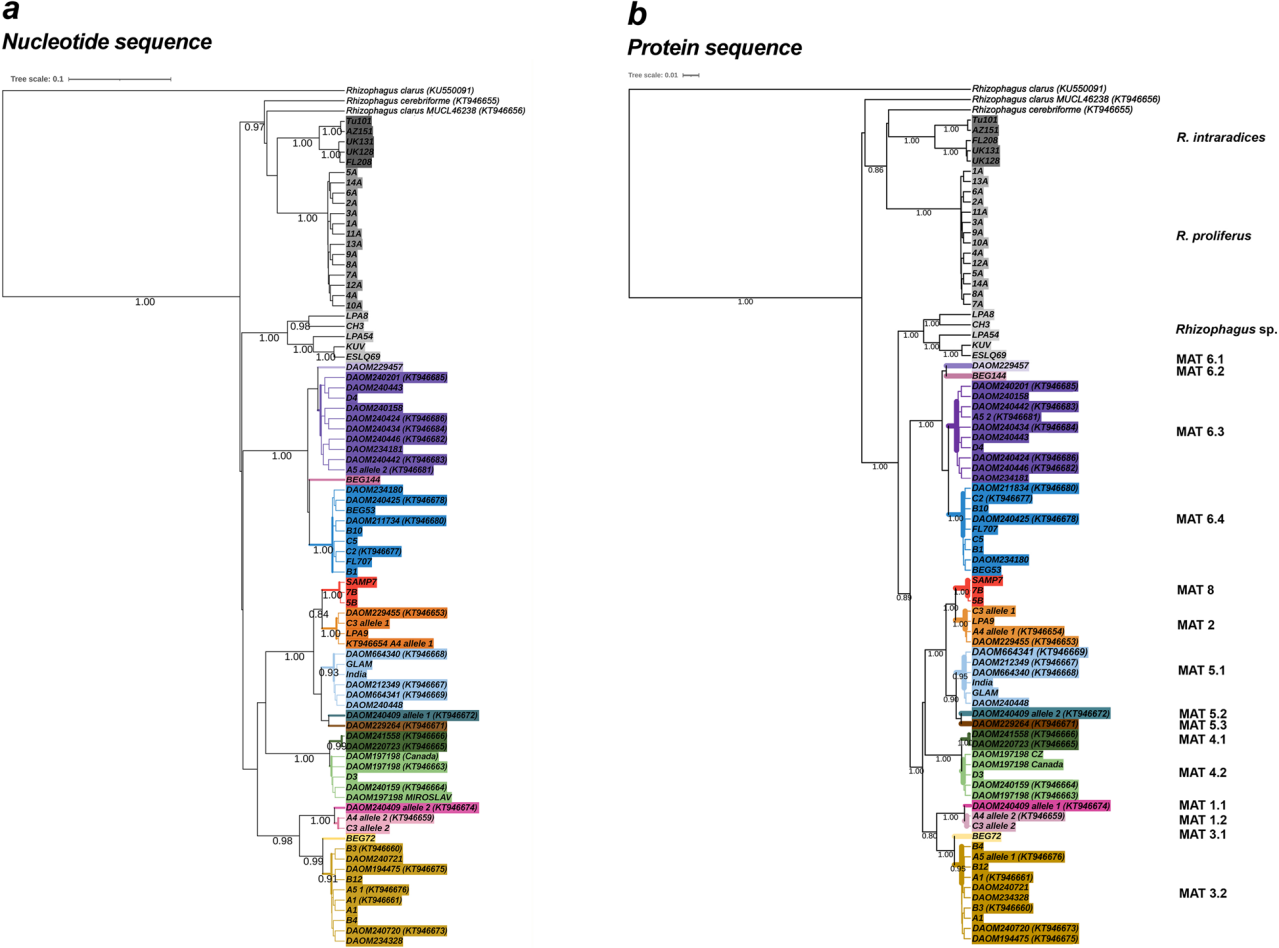
A Bayesian phylogeny based on MAT-types of 81 sequences of *Rhizophagus spp* and isolates. MAT-types in the main *R. irregularis* cluster are further divided into different MAT-types, where the first number before the decimal point represents the MAT-types defined by previous studies [14, 28] and where the number after the decimal point represents strongly supported divergence within a given MAT-type. MAT-type 8 is newly identified in this study. Text in parentheses following the name of an isolate represents the Genbank accession code for sequences used from previous publications [14, 36]. Both the high posterior probability values (> 0.9) and internal reference sequences (recognisable by the Genbank accession code in parentheses) from were used to verify different *Rhizophagus* species and MAT-types reported in previous studies [14, 28]. The sequence of *Rhizophagus clarus* (KU550091) served as a root for the tree. Each cluster was assigned a specific colour, while the groups that are not *R. irregularis* were displayed in different shades of grey. The revealed MAT-types were not only supported by nucleotide sequence divergence (**a**), but also by amino acid sequence divergence (**b**)

Based on sequence divergence at the MAT-locus, the *Rhizophagus* isolates were grouped into distinctive MAT-types (Fig. 2a). We found the MAT phylogeny discriminated different species of the *Rhizophagus* genus. The phylogeny showed a clear clustering of *R. intraradices* isolates, supported with a high posterior probability of 1.00. Another well supported group, *R. proliferus,* clustered with *R. intraradices* with a posterior probability of 1.00. This is in congruence with previous publication based on double digest restriction-site associated DNA (ddRAD) sequencing [27]. Overall, the sequences at the MAT-locus were able to assess the interspecific diversity within the *Rhizophagus* genus and discriminate potentially diverged isolates within a group. Interestingly, isolates LPA54, ESQLS69 and KUVA were previously reported as *R. irregularis* by Savary et al. [27], although their genomes showed divergence from the main genetic group of *R. irregularis*. Even though the MAT-types of these isolates showed higher similarity for the *R. irregularis* group by forming a monophyletic cluster, the group was still separated from main *R. irregularis* group with high posterior probability (1.00). Each of the five isolates of the *Rhizophagus* sp. were different from each other, even affecting the amino acid sequences of their MAT-loci in coding region of HD2 protein (Figure S1). The result clearly indicates several different MAT-types among the isolates of this group.

### MAT-type diversity in *R. irregularis* is higher than expected

Our initial assignment of MAT-types to each *R. irregularis* isolate was based on clustering of the seven previously published MAT-types. Each MAT-type was defined by a custumal threshold of posterior probability value, ranging between 0.90 and 1.00 (Fig. 2a). Using this procedure, we observed 7 main clusters representing 6 of the 7 previously identified MAT types and an 8th previously unreported MAT-type. This newly identified MAT-type showed closest sequence similarity to MAT2 and is labelled here as MAT8. However, we found considerable sequence divergence within some of the main MAT-types observed in this study. The majority of sequence divergences within a MAT-type represented non-synonymous mutations and was directly linked to amino acid sequence divergence of the HD2-encoding protein; (Figure S2b). We, thus, defined different alleles within each MAT-type as those containing substitutions that altered the amino acid sequence. Subdivisions, based on non-synonymous substitutions, were observed in MAT-types 1, 3, 4, 5 and 6. Two sub-groups were observed in MAT1 (MAT1.1 and MAT1.2), MAT3 (MAT3.1 and MAT3.2) and MAT4 (MAT4.1 and MAT4.2). This divergence was supported by high posterior probability values and sequence divergence. MAT5 was divided into three sub-groups having the respective MAT-types (MAT5.1, MAT5.2 and MAT5.3). Lastly, MAT6 was further separated into four MAT-types, referred to here as MAT6.1 to MAT6.4 (Figure S2). In summary, *R. irregularis* displayed an unexpected diversity of MAT-types, based on amino acid and DNA sequence divergence, with 15 different alleles.

### MAT-locus and PTG phylogenies are congruent with both loci under purifying selection and codon usage similarity

**Fig. 3:**
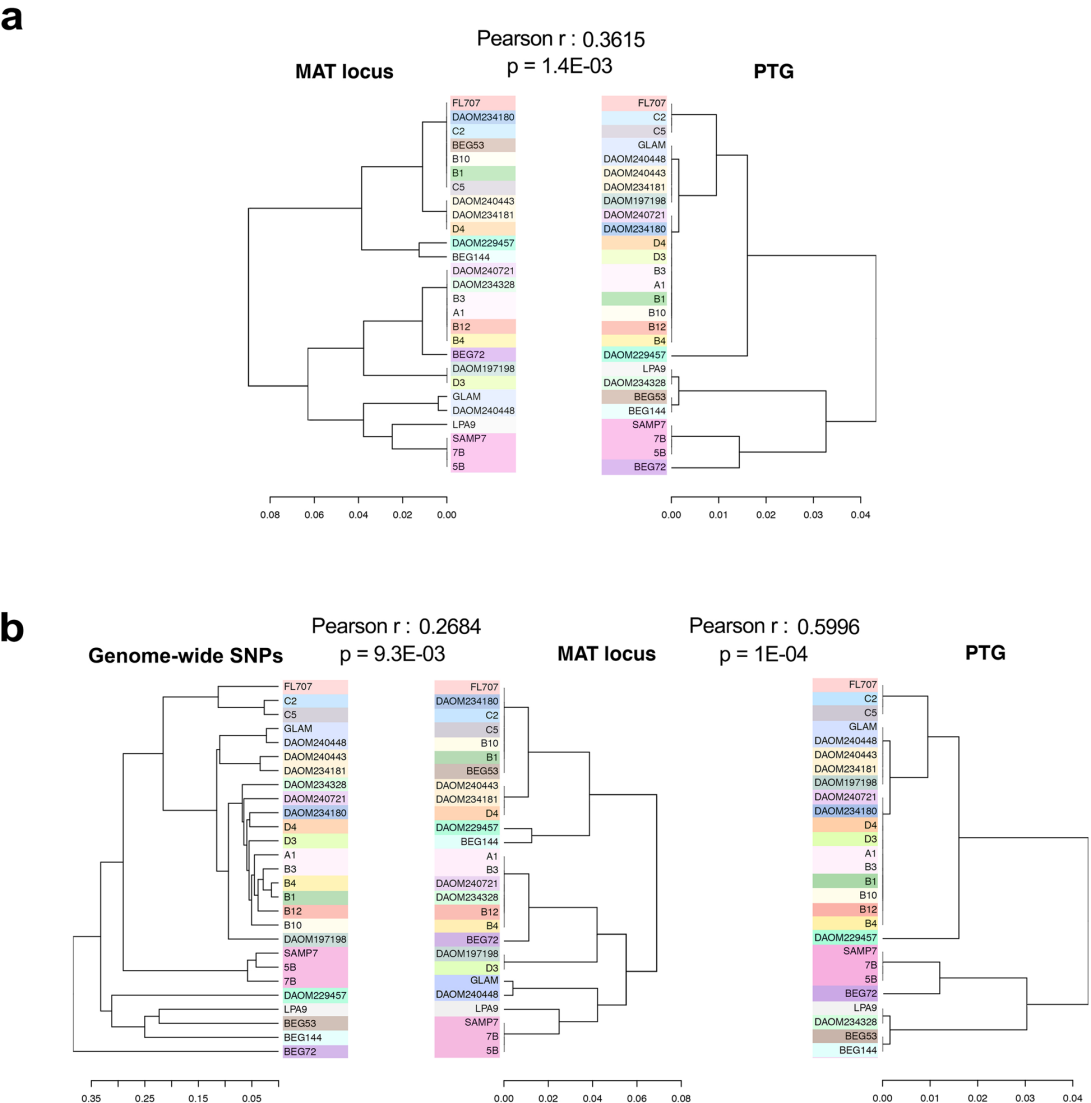
Congruence among three phylogenetic trees based on genome-wide SNPs, the MAT-locus and PTG in *R. irregularis*. Distances among isolates based on genome-wide data were calculated using data on 47,378 SNPs without locus overlap with the MAT-locus or PTG. Distances of divergence in partial MAT-locus (261 bp) and PTG (639 bp) were calculated by Tamura 3-parameter model [37]. Matching nodes or clusters between phylogenies are highlighted in the same colour. **a** Significant correlation of both MAT-locus and PTG phylogenies. **b** Significant correlation of phylogeny from genome-wide SNPs to MAT-locus and to the PTG phylogeny

In a scenario of sexual reproduction, a given gene, or a randomly selected coding region, is unlikely to evolve together with another locus, especially when the two loci are not functionally linked [30]. We tested the linear correlation between the divergence of the MAT and PTG loci. Unexpectedly, a significant correlation was observed (Mantel test; *ρ* = 0.7447, *p* < 1E^−04^) between divergence of alleles at the MAT and PTG loci in data comprising all *Rhizophagus* species and isolates (*N* = 37). The linear correlation was also significant when we tested for a correlation between intraspecific divergence in the MAT and PTG loci in *R. irregularis* (*N* = 30) (Mantel test; *ρ* = 0.4076, *p* < 7E^−04^), Because some isolates that were previously described as *R. irregularis* grouped outside this species in the MAT phylogeny (Fig. 2), we also performed Mantel test after excluding those isolates from the analysis. This more conservative Mantel test was also significant showing high correlation between the MAT and PTG phylogenies (*N* = 27) (Mantel test; *ρ* = 0.3615, *p* < 1.4E^−03^). All correlations were positive, meaning that more divergence in MAT-locus is associated with more divergence in the PTG. A further test of congruency between the phylogenies, using Baker’s gamma, was performed with the most conservative grouping that only included *R. irregularis* isolates that clustered as *R. irregularis* in the MAT phylogeny. This also showed significant similarity between MAT-locus and PTG phylogenies (*p* = 0.032) (Figs. 3a and S3a).

**Fig. 4:**
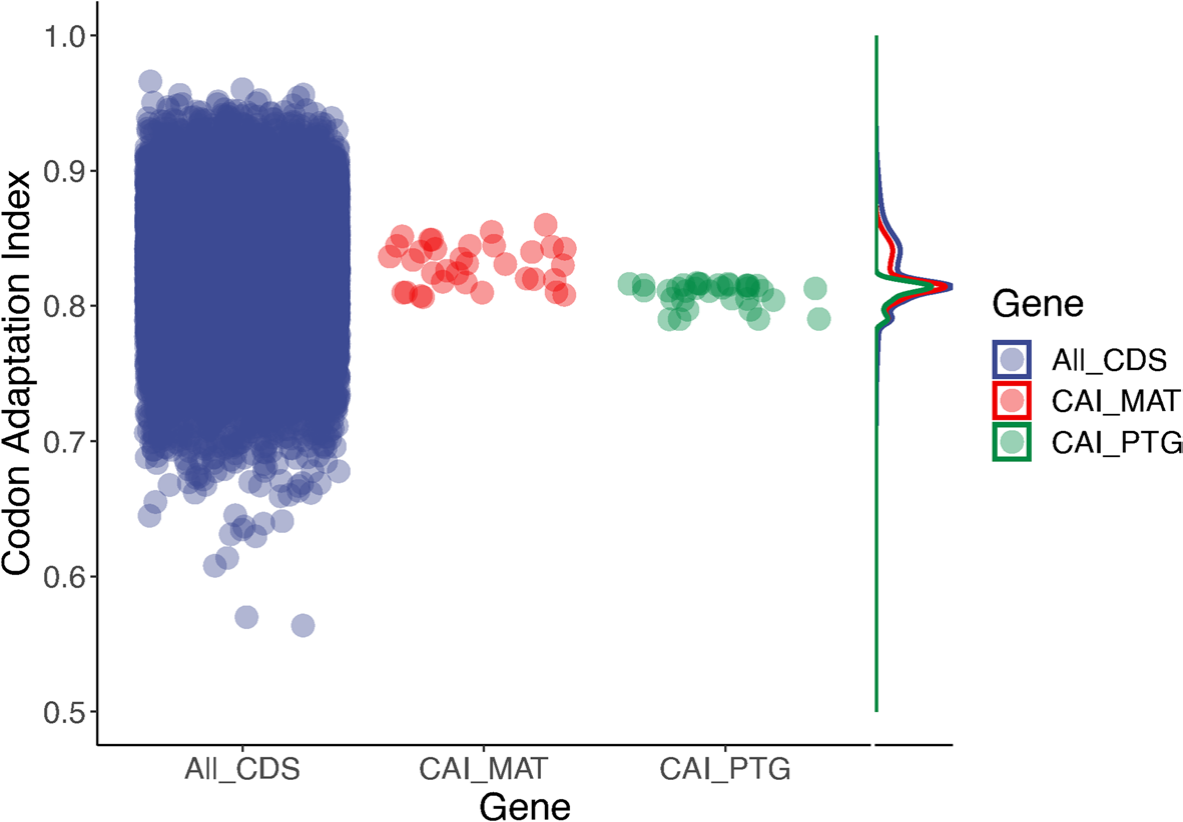
Codon adaptation index (CAI) analysis reveals genome-wide codon usage similarity in the reference isolate (DAOM197198) and the among-isolate codon usage similarity of the MAT-locus and PTG. All CDS (blue) represents CAI from a total of 26,183 genes in the reference isolate (DAOM197198). CAI_MAT (red) and CAI_PTG (green) represent the CAI of the target gene in each isolate, respectively. Observed high CAI in all datasets (distribution plot peak at CAI > 0.8) and clear overlap of the peaks among datasets show the both MAT-locus and PTG of different isolate have similar codon usage and the CAI of the two genes are not the exceptions to overall converged codon usage detected in the reference isolate

To answer whether the observed correlation between the MAT-locus and PTG divergence are likely to be a result of purifying or positive selection, we tested codon evolution in both the MAT-locus and PTG (Table 1). Purifying selection removes deleterious variations and, therefore, contributes to the functional stability of a gene. On the other hand, positive selection promotes the spread of beneficial alleles and contributes to the gene diversity for environmental adaptation [38]. Both loci were shown to have evolved under purifying selection (Table 1), allowing us to reject the hypothesis that they were under positive selection. Together with the observed high diversity of MAT-alleles carrying non-synonymous mutations, the results implied the diversification of MAT-alleles still occurred under purifying selection. Additionally, both loci also showed high codon usage similarity, between loci, as well as among the isolates (MAT-locus average codon adaptation index (CAI): 0.830, expected value of CAI (eCAI): 0.857 (*P* < 0.05) and PTG average CAI: 0.809, eCAI: 0.821 (*P* < 0.05)) (Fig. 4). The observed inter-isolate CAI of the MAT-locus and PTG had an overlapping distribution peak (Fig. 4) which also overlapped perfectly with the intra-isolate CAI distribution peak of 26,183 genes in the reference isolate (DAOM197198; average CAI: 0.818, eCAI: 0.862; *P* < 0.05). This showed that codon usage convergence in these two genes is not an exception compared to the overall observed genome-wide codon usage similarities in *R. irregularis*. Our results suggest that even though the two loci are not functionally related, both MAT-locus and PTG evolution were driven by purifying selection resulting the converged codon usage similarity and their correlated divergence.

**Table 1:** Codon-based test of selection averaged over all sequence pairs of the PTG and MAT-locus. Z-test was conducted using the Nei-Gojobori method [39]. In the test, d_N_ and d_S_ are the numbers of non-synonymous and synonymous substitutions per site, respectively. 999 bootstrap replicates were applied to test significance. The value of d_S_ is always larger than d_N_, consistent with the purifying selection observed in PTG and MAT-locus

Target	d_N_	d_S_	Test type	Significance	Z-test statistics
Phosphate transporter (PTG) alleles	0.0293	0.1717	Positive selection	1	-7.9443
			Purifying selection	< 0.001	
MAT alleles (partial HD2)	0.0508	0.1140	Positive selection	1	-2.3788
			Purifying selection	0.0109

### Genome divergence is congruent with both MAT-locus and PTG divergence

The ddRAD-seq dataset provided us with a total of 47,378 SNPs, representing random coding and non-coding regions of the *R. irregularis* genome. By only focusing on homokaryons, which avoids potential single nucleotide polymorphisms (SNP) calling bias incurred by dikaryons, and by having a dataset with over six times more SNPs than previous population studies [27], we were able to achieve a higher resolution of genome diversity in *R. irregularis* (Fig. 3b). Geographic origin could not explain the similarities among genomes (PERMANOVA with Jaccard distance, *p* > 0.05), but isolate MAT-type explained genome similarity (PERMANOVA with Jaccard distance, *p* < 0.05). A Mantel test was performed to examine the correlation. We found significant correlation between the divergence of genomes and the divergence of the MAT-locus which implies co-evolution or associated evolution of the sets of random coding and non-coding regions of the genome together with the MAT-locus (*N* = 37) (Mantel test; *ρ* = 0.4601, *p* < 1E^−04^). The correlation was positive, indicating that greater divergence in genomes was reflected in greater divergence in their MAT alleles. The significance of observed correlation of the entire dataset was not influenced by additional filtering for isolate selection, showing the correlation holds true at both interspecific and intraspecific levels. A significant correlation also occurred within *R. irregularis* isolates (*N* = 30) (Mantel test; *ρ* = 0.3303, *p* = 0.0067) as well as the more conservative group excluding isolates that clustered outside the *R. irregularis* group of the MAT locus phylogeny (*N* = 27) (Mantel test; *ρ* = 0.2684, *p* = 0.0093). With this more conservative group of *R. irregularis* isolates we also found a linear correlation between the distance among genome-wide SNPs and the PTG divergence (Pearson *ρ* = 0.5996, *p* = 0.0044). Together with the previously described positive linear correlation between PTG and MAT sequence divergence, all 3 possible pairwise comparisons among genome-wide SNPs, MAT-locus and PTG distance matrices of *R. irregularis* showed significant correlation. The results are also congruent with the detected intra- and inter-isolate levels of codon usage similarities (Fig. 4). Further analysis of phylogenetic congruency with Baker’s gamma among these three datasets also confirmed the significant phylogenetic congruency observed in all pairwise correlations among genome-wide SNPs, MAT-locus and PTG (*p* < 0.05 in all comparisons) (Fig. 3 and S3).

## Discussion

In this study, we found that the model AMF, *R. irregularis,* harbours diversity at the MAT-locus that is higher than that expected for a fungus that could, at most, exhibit facultative or rare sexual reproduction. This result, on its own, would signify that either this locus is not involved in sex, or that extremely frequent sex has maintained its diversity. However, we also tested whether *R. irregularis* is likely a sexually reproducing species, based on the fundamental evolutionary concept of decoupling between the evolutionary history of the MAT-locus, another locus independent of mating and genome-wide arbitrarily chosen loci [30]. In *R. irregularis*, the congruence between the evolutionary history of these three datasets, and including codon usage, matches that of a clonal organism, and thus contradicts the notion that very frequent sex to maintain diversity at the MAT-locus has occurred. Taken together, these results allow us to reject the hypothesis of sexual reproduction in this important fungal species (Fig. 1c). Credence to the hypotheses that AMF are sexual is strongly based on the existence of the MAT-locus in AMF and that dikaryons carry two different copies. The results of this study suggest that it is highly unlikely that this species is sexually reproducing. Our findings have a number of important consequences that we discuss in more detail.

### Variation in the *R. irregularis* MAT-locus is not consistent with the MAT-locus being involved in mating

Recent studies of sexuality and potential recombination in AMF have relied on detecting recombination between genomes carrying different MAT alleles. Seven MAT-types had been reported in *R. irregularis* which suggested possible cryptic, but active sex and recombination [28]. In that study, the approach to define different MAT-types was based on phylogenetic analyses of MAT-locus sequence similarity with a defined node-support value, or threshold, for each cluster. However, the posterior probability values for the nodes depend on the relative similarity between MAT-locus sequences and do not reflect the true sequence difference. We found that nucleotide sequence variation at the MAT-locus affects the HD2 amino acid sequence; a homeodomain transcription factor that plays an important role in self/non-self recognition in other fungi [16]. If the sequence at the *R. irregularis* MAT-locus defines mating types in this species, the sequences should be highly conserved as is the case for other fungi [34]. Relying on an arbitrary threshold of node support to define MAT-type clusters overlooks the actual diversity of existing MAT-types. Previous studies targeted a less variable region of the locus. and this likely hindered finding true MAT-locus variation. We found 15 MAT types out of 50 unambiguous *R. irregularis* isolates. The frequency (approx. 0.3) is almost five time higher than that previously reported (0.061; 7 MAT-types in 114 isolates). However, several of the 50 isolates in our study are undistinguishable from each other with ddRAD-seq data. Thus, it is likely that the 15 MAT-types occur in considerably less than 50 genetically different *R. irregularis* isolates. Furthermore, our study and previous studies only considered partial sequences at the MAT-locus. Full length sequences could contain more nucleotide sequence variation. To maintain such a number of MAT-alleles, the species should undergo frequent sex as in the cases of other fungal species [15, 19–21]. We conclude that the higher-than-expected level of MAT-type variation is inconsistent with the proposed cryptic rare sexuality in these fungi. The alternative explanation is that the MAT-locus which fundamentally constitutes the current sexuality paradigm of AMF, is not involved in mating.

### The co-evolution of genome-wide variation with PTG, and the MAT-locus variation points strongly to asexuality

Mating, followed by recombination, decouple the evolution of different loci in the population. In contrast, loci of asexual organisms share the same genealogical history [30]. If *R. irregularis* is sexual and recombination takes place, it is highly unlikely that the intraspecific diversification of two non-related loci is linked to the evolutionary history of the entire genome. The PTG is an important AMF gene, as the symbiosis between plants and AMF is constituted by the nutrient exchange, especially translocation of soil phosphate from the fungus to the plant [1]. The genes of MAT-locus and PTG were known to have clearly different functions [16, 40]. The two loci are located on two different chromosomes (chromosome 11 for HD2 and 18 for PTG in *R. irregularis* DAOM197198) [41]. Strikingly, we found a clear positive linear correlation in the divergence between these two functionally and distantly unrelated loci, which is expected in the absence of recombination. Moreover, we found that the sequence divergence, based on multiple genome-wide coding and non-coding regions across the *R. irregularis* genome, and in which both the MAT-locus and PTG were excluded, was congruent with the MAT-locus or PTG phylogenies. The observed congruency in sequence divergence is further supported by the clear codon usage similarities between the MAT-locus and PTG. However, more surprisingly, the known coding regions of the reference isolate genome showed strong genome-wide codon usage convergence which is consistent with the correlated genealogical history among different loci in the genome. The fact that the MAT-locus, PTG and *R. irregularis* genome (excluding the MAT-locus and PTG) are correlated, and share similar codon usage, represents a genomic feature which consistent with a clonal organism.

### Should we be searching for alternative MAT-loci in AMF and what are the possible roles of the locus?

The conserved putative MAT-locus reported in *Rhizophagus* spp. is the homolog of a fungal mating type locus that is conserved in Basidiomycete fungi. From the evidence presented here, it seems highly unlikely that this putative MAT-locus is involved in mating in AMF. Some other known genes of the Dikarya or Mucoromycota (ancient and recent fungal lineages) that are involved in mating and recognition are present in AMF genomes [11] and are expressed during co-inoculation of roots with two genetically different *R. irregularis* isolates [42, 43]. However, those genes are not all fully conserved, and most also have other known functions in fungi (for example, conidia development and germination, mycotoxin production and oxidative stress response) [22, 44] and none of them have two different alleles in dikaryons. For these reasons, they are unlikely candidates as MAT-loci in *R. irregularis*. Thus, while *R. irregularis* genomic features do not point to sexuality, if endeavours towards finding a true MAT-locus in AMF continue then we propose two additional important criteria that must be satisfied. First, it will be a previously undescribed locus in the fungal kingdom. Second, the locus should display a very low degree of allele diversity as frequent sex in this fungus can be excluded.

So, what is the role of the MAT-locus studied here? One possibility is that *R. irregularis* was very active sexually in the past, giving rise to high MAT-type diversity, and at a certain point in the evolution of the lineage, the fungi lost the ability to sexually reproduce. However, this locus still contains a conserved HD1-like and HD2 protein. MAT-loci identified in other asexual fungi were found to be involved in asexual functions, such as asexual sporulation and cell cycle regulation [22]. It is well known that *R. irregularis* anastomoses with hyphae of the same genotype and that four different stages of recognition and compatibility between pairs of genetically different *R. irregularis* isolates have been described [12]. In the case of successful fusion, this allows cytoplasm of the two individuals to flow rapidly in both directions [12]. It is conceivable that the locus could be involved in some of these recognition mechanisms allowing, or preventing, the fusion of hyphae of compatible individuals to distribute nutrients and improve structural integrity of hyphal networks. This could also allow the co-existence of genetically different multiple nuclei in one cytoplasm, even if no recombination takes place between them.

### The asexual *Rhizophagus irregularis* lineage does not mean Glomeromycota are ancient asexuals

Explaining the existence of ancient asexual lineages is problematic in evolutionary biology because a fundamental role of recombination is to purge deleterious mutations [45]. The Glomeromycota are thought to be an ancient lineage that formed symbioses with plants since the colonisation of land. Coupled with their seemingly low morphological diversification, they were suggested to be ancient asexuals [9]. The fossil record for Glomeromycota is extremely poor and there could have been great diversification in the Glomeromycota in the past, as seen for the species diversity of major plant lineages that preceded angiosperm radiation. There is a danger that our results on the asexuality of *R. irregularis* will be interpreted as evidence for the long-term asexuality of the Glomeromycota lineage. While our results strongly support asexuality in *R. irregularis*, we are not claiming the whole Glomeromycota lineage to be asexual. While there are hardly any confirmed examples of long-lived asexual lineages, there are many examples in nature where an order or genus contains sexual and asexual species [46]. This may be the case in the Glomeromycota. To answer the separate question of sexuality *versus* asexuality in the Glomeromycota lineage, we urge researchers to carry out similar studies on other Glomeromycota species spread widely across the phylogeny.

### Exciting consequences for AMF applications in agriculture and the environment

The question of sexuality in *R. irregularis* will greatly affect how AMF can be applied to improve agricultural production and ecosystem functions. Our findings may disappoint researchers intending to develop a breeding program relying on crossing to improve AMF. Our results show that this will likely not be possible with *R. irregularis*. However, other genetic mechanisms in *R. irregularis* allow the development of new strains of this fungus that have been shown to greatly alter productivity of globally important crops [5, 8, 47].

However, there are positive consequences of our findings. First, *R. irregularis* is a safe AMF species to develop for agricultural applications because it can be produced readily in vitro without other unwanted microorganisms. That introduced an *R. irregularis* strain will not recombine with local AMF populations is of great benefit for applications because there should be no introgression of introduced genes into the local population and the introduced fungus should retain its functional characteristics. Secondly, there is no current method to track in introduced *R. irregularis* in soil where *Rhizopagus* spp already occur (which is usually the case in agricultural soils). Each nucleus of *R. irregularis* is haploid and, thus, represents the genome of the individual and all nuclei in a homokaryon individual are identical. Each nucleus carries one MAT-type. Because MAT-type variation is positively correlated with variation in the *R. irregularis* genome, MAT-type variation represents an excellent proxy for studying *Rhizophagus* variation in populations, which was previously not possible. This will allow direct tracking of introduced *Rhizophagus* to finally allow the study of AMF invasiveness. Furthermore, it will be the first time researchers have a tool for directly studying the population biology of this important fungus to measure diversity, and to allow the study AMF competition and co-existence.

## Materials and Methods

### Fungal isolates included in the study of MAT-locus diversity

A total of 51 isolates of *Rhizophagus* species (comprising, *R. clarus*, *R. cerebriforme*, *R. irregularis*, *R. intraradices*, *R. proliferus*, and an undescribed *Rhizophagus* species) were used in the study for sequencing of the MAT-locus. The isolates originated from the soils of 13 countries in four continents and were isolated between 1981 and 2013 (Table S1). All the isolates were maintained as monoxenic in vitro cultures with root inducing (Ri) T-DNA transformed carrot (*Daucus carota*) roots in Petri dishes containing minimal (M) medium solidified with 0.4% phytagel [48]. All in vitro cultures were initiated from a single spore. The cultures were incubated at 25 °C under dark for 12 weeks.

### Collection of fungal material, DNA extraction, purification, and quantification

For collection of fungal material for DNA extraction, citrate buffer was first used to dissolve the medium [35]. After dissolving the medium, the mycelium was collected, washed with the MiliQ water three times. Samples were then immediately frozen with liquid nitrogen and stored at -80 °C until DNA extraction. The DNA was extracted with DNeasy® Plant Mini Kit (Qiagen, Switzerland), following manufacturer’s protocol. After extraction, DNA samples were purified using the Monarch PCR & DNA Cleanup Kit (New England BioLabs, United States) and further quantified using Qubit™ dsDNA HS assay kit (ThermoFisher Scientific, Switzerland). After quantification, all DNA samples were diluted with MiliQ water to obtain a final concentration of 2 ng·µL^−1^.

### Designing degenerate primers to target a variable region of MAT locus

The full length MAT locus in AMF genomes from previously published studies [14, 36] were retrieved and aligned by using MUSCLE v5 [49]. Based on multiple sequence alignment (MSA), regions were surveyed for genetic variability. Degenerate primers (forward primer (SJF): 5’-CGTGRGCGKATTACCAAGGA-3’ and reverse primer (SJR): 5’-GACATGGTTCAATAATAGAAGAAATCG-3’) were designed manually to yield an approximately 300 bp amplicon length (Table S2). The primers were tested in silico with IDT OligoAnalyzer (https://eu.idtdna.com/pages/tools/oligoanalyzer) for the T_m_ and potential homo- and hetero-dimer formation. Target specificity was also tested and confirmed by the National Center for Biotechnology Information (NCBI) PrimerBLAST with a targeted search against a non-redundant sequence database (NR).

### DNA amplification and sequencing of the MAT locus

Polymerase chain reaction (PCR) was conducted with Taq PCR Master Mix (Qiagen, Switzerland). Total reaction volume was 20 µL with 2 × Taq PCR Master Mix, 2 µL of primer pair (1 µM) and 4 ng of template DNA. Amplifications were performed in SimpliAmpTM Thermal Cycler (Applied biosystems, Switzerland) with 32 cycles of 1 min at 94 °C, 45 s at 55 °C, and 1 min at 72 °C, followed by a final extension step of 10 min at 72 °C. PCR amplicons were purified using the Monarch PCR & DNA Cleanup Kit (New England Biolabs, United States) and quantified using a Qubit™ dsDNA HS assay kit (ThermoFisher Scientific, Switzerland. The purified amplicons were diluted to obtain a final concentration of 8 ng/µL and pair-end sequenced using Sanger sequencing technology with two technical replicates and two biological replicates of each isolate. The sequences were deposited in the International Nucleotide Sequence Database Collaboration (INSDC) and publicly available at through the NCBI Genbank at (https://www.ncbi.nlm.nih.gov/nuccore/) under accession codes: LC738554 to LC738607.

### Phylogenetic analysis of MAT-locus

Sequences of the MAT locus from 51 homokaryon isolates of present study and 30 publicly available MAT sequences of homokaryons and dikaryons from previous studies [14, 36] were aligned using MUSCLE v5 [49]. After trimming, the 81 sequences with a 281 bp length were subjected to model testing for Bayesian phylogenetic analysis by JModelTest v2.1.1036 [50]. The resulting best nucleotide substitution model was the Hasegawa-Kishino-Yano (HKY) model with a gamma distribution. To test the protein coding gene divergence, translated amino acid sequences of the partial HD2 gene, covered by sequenced amplicons. The protein evolution model test was conducted on a multiple sequence alignment of 81 amino acids with ProTest v3.4.2 [51]. The selected best nucleotide substitution model was the Jones-Taylor-Thornton (JTT) model with a gamma distribution. The Bayesian phylogenetic analyses were conducted with BEAST2.5 [52], with 10,000,000 generations and with a burning in of the first 20% generations. The resulting phylogenies were visualised using iTOL (https://itol.embl.de).

### Building pairwise distance matrices of MAT-types and phosphate transporter gene (PTG) alleles and calculation of codon usage index

Publicly available phosphate transporter gene (PTG) sequences of homokaryon isolates were retrieved if corresponding genome-wide SNP data were also available [27]. The sequences from a total of 37 isolates were retrieved and used for downstream analyses. Pairwise distance matrices of the published PTG sequences and the corresponding MAT sequences of current study were built using the pairwise nucleotide sequence similarity calculated with the Tamura 3-parameter model [37]. Codon evolution of MAT alleles and PTG alleles was also tested by analysing the numbers of nonsynonymous (d_N_), synonymous (d_S_) substitutions and their variances: Var(d_N_) and Var(d_S_). Analyses were conducted using the Nei-Gojobori method [39]. The Z-value was used for testing the null hypothesis: Z = (d_N_—d_S_) / SQRT (Var(d_N_) + Var(d_S_)) The null hypothesis (H_0_) for the tests of positive or negative selection was: There is no difference between strict-neutrality (d_N_ = d_S_, Z = 0). The threshold to reject the null hypothesis was set to 0.05. Synonymous codon usage bias among the isolates was measured by calculating the codon adaptation index (CAI) in MAT and PTG sequences by CAIcal [53]. For the intra-isolate codon usage bias calculation, the coding sequences of a total 26,183 genes in the model *R. irregularis* isolate (DAOM197198) were used as a reference.

### Building a genome-wide SNP database

To compare the overall genome variation of each isolate with the variation in PTG and at the MAT sequences, we built genome-wide SNP database. Raw reads of homokaryon isolates from two previous studies using double digest restriction-site associated DNA sequencing (ddRAD-seq; [27, 35] were downloaded from NCBI and analysed using Stacks v2.3 [54]. We downloaded data for the same 37 isolates used for the building pairwise distance matrices of MAT-types and PTG. However, for the accurate calculation of the genetic differences among isolates, we removed the data of any isolates that were considered ambiguous*.* By ambiguous, we mean those isolates that were previously assigned to *R. irregularis* according to Savary et al. [27], but that did not cluster with *R. irregularis* according to the MAT or PTG phylogenies of this study. For the calculation of single nucleotide polymorphisms (SNP) across the isolates, we did not include any data of dikaryons from previous studies, as they contain mixed reads originating from two different genomes. After excluding ambiguous isolates, 27 *R. irregularis* homokaryons were retained that could be compared with both MAT and PTG sequences. Low quality reads were trimmed with PrinSeq-lite 0.20.4 lite [55] with default parameters. Demultiplexing of sequences was performed using Stacks command “process_radtags”. Demultiplexed sequences from homokaryon isolates were mapped to the version 2.0 genome of *R. irregularis* DAOM197198 from Joint genome institute (JGI) [56] as reference using Burrows-Wheeler Alignment tool (BWA) v0.7.17 [57], with the default parameters of “bwa mem”. The obtained.sam files were then converted into.bam files via SAMtools v1.1043 [58]. SNP calling at each locus was performed using the gstacks from Stacks [54] and exported as.genepop files using the command “populations –genepop”. The –min-mapq gstacks parameter for minimum PHRED-scaled mapping quality was set at 60 and population parameters for minimum allele counts required to process a SNP was set to 2 with –min-mac and the observed heterozygosity was set to 0 with –max-obs-het. The resulting.genepop files were converted into.gen. All above described analyses were performed on the high performance computing server of the University of Lausanne, Switzerland. The generated SNP data of the isolates were further converted into.genind object using the Adegenet package [59] in R v4.0.0 [60]. Further filtering was applied to the SNP dataset. SNPs were located in coding and non-coding regions and contained at least 10 reads of coverage. Only SNPs supported by more than 80% reads of each isolate were considered. The SNPs in the MAT locus and PTG encoding regions were removed to avoid the effect of sequence divergence of those loci on the genome similarity/dissimilarity calculation. This was necessary for subsequent tests for congruence between phylogenies generated using the SNP database and the phylogenies of the PTG and MAT-locus.

### Statistical analysis

A Jaccard distance matrix of isolate based on the genome-wide SNP data was computed with “vegdist” from the *vegan* R package [61]. Permutational multivariate analysis of variance (PERMANOVA), with “adonis” was computed to test the clustering by geographic origin or MAT-type. The tested null hypothesis (H_0_) was: There is no difference in nucleus genotype clustering by geographic origin or MAT-type. The genome-wide phylogeny based on hierarchical clustering analysis was performed using the R package *pvclust* [62]. The R package *ggplot2* [63] was used for graphic visualisation of plots. These were further modified with the software Inkscape v1.1 for adding related metadata. To test the correlations among the divergences of genomes, MAT-locus and PTG in distance matrix level, pairwise Mantel tests were applied with vegan package [61] in R v4.0.0 [60]. The null hypothesis (H_0_) tested was: There is no linear correlation between pairs of matrices. To test the congruency of genome, MAT-locus and PTG divergence, dendrograms were built from the corresponding distance matrices with Ward clustering option in *dendextend* R package [64]. Following pairwise Baker’s gamma [65] was calculated and statistical significance was tested with 1000 permutations. The null hypothesis (H_0_) tested was: There is no association between the two phylogenetic trees. For the test of codon preferences of MAT-locus and PTG, as well as all coding sequences in the reference isolate (DAOM197198), the expected value of the codon adaptation index (eCAI) was calculated by E-CAI [53]. The null H_0_ tested was: Measured eCAI are artefacts that arise from internal biases in the G + C composition and/or amino acid composition of the target sequences. In all statistical analyses, the threshold to reject the null hypothesis was set to 0.05.

## Supplementary Information

Supplementary Material 1.

Supplementary Material 2.

Supplementary Material 3.

Supplementary Material 4.

## Data Availability

The data generated and/or analysed during the current study are deposited in the International Nucleotide Sequence Database Collaboration (INSDC) and publicly available at through the NCBI Genbank at (https://www.ncbi.nlm.nih.gov/nuccore/) under accession codes: LC738554 to LC738607.

## Abbreviations

AMF: Arbuscular mycorrhizal fungi
ddRAD: Double digest restriction-site associated DNA
MAT locus: Mating-type locus
PTG: Phosphate transporter gene
SNP: Single nucleotide polymorphism
