# Supplementary figures and images for: Evolution of unexpected diversity in a putative mating type locus and its correlation with genome variability reveals likely asexuality in the model mycorrhizal fungus Rhizophagus irregularis
Source: BMC Genomics. 2024 Sep 20;25:888. doi: 10.1186/s12864-024-10770-9 (PMC11414155; doi:10.1186/s12864-024-10770-9)

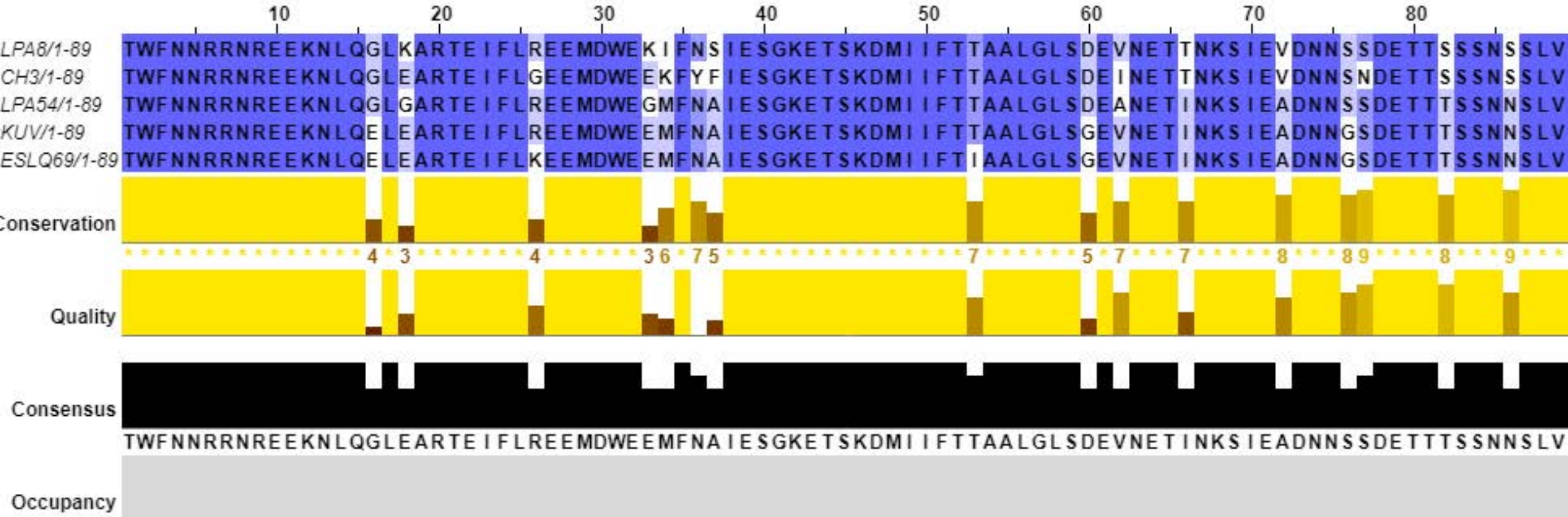

Supplement: Supplementary file 1 — Supplementary Material 1. [file 12864_2024_10770_MOESM1_ESM.pdf]

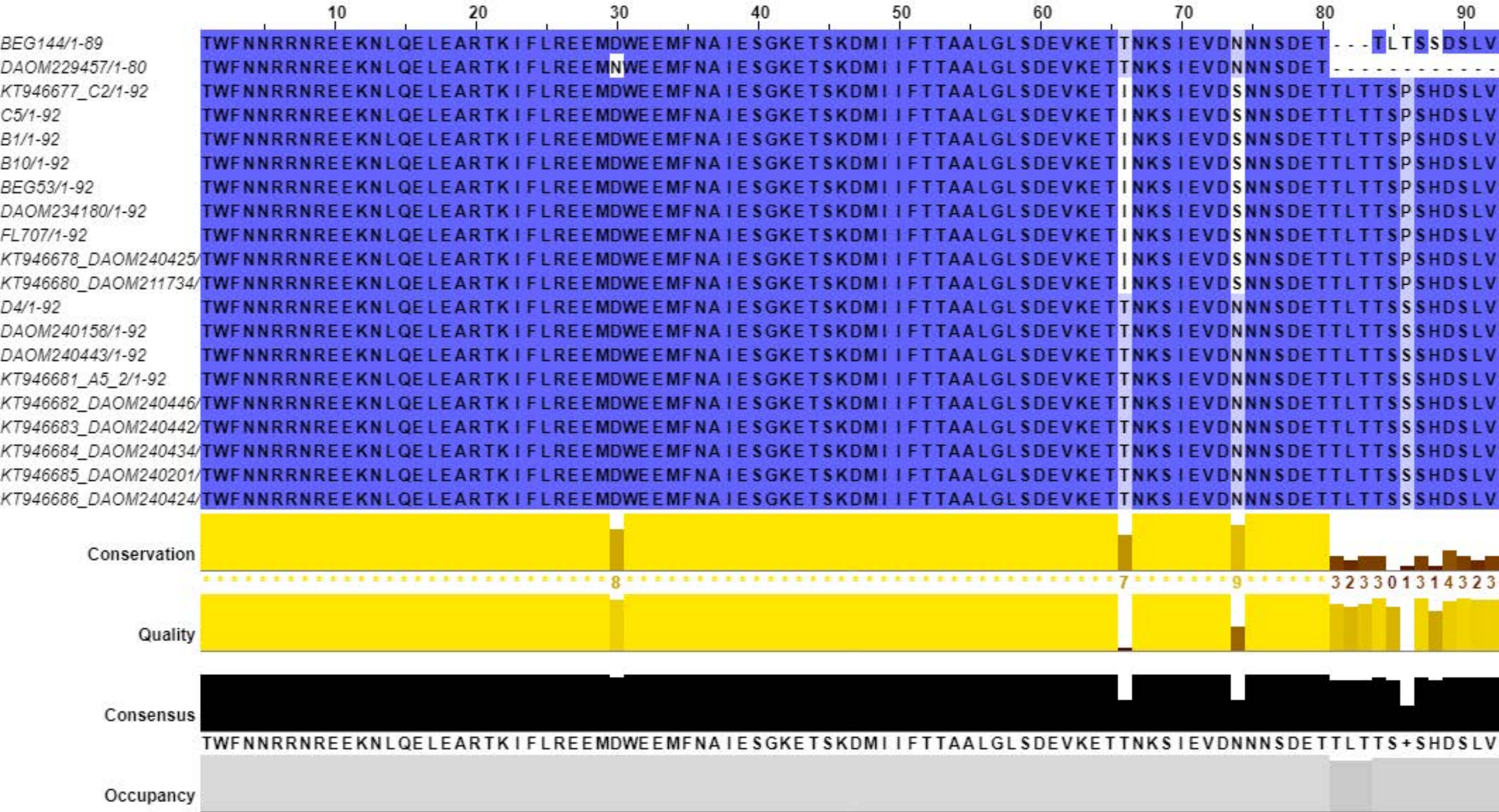

Supplement: Supplementary file 2 — Supplementary Material 2. [file 12864_2024_10770_MOESM2_ESM.pdf]
