# Supplementary material for: Evolution of unexpected diversity in a putative mating type locus and its correlation with genome variability reveals likely asexuality in the model mycorrhizal fungus Rhizophagus irregularis
Source: BMC Genomics. 2024 Sep 20;25:888. doi: 10.1186/s12864-024-10770-9 (PMC11414155; doi:10.1186/s12864-024-10770-9)
Supplement: Supplementary file 3 — Supplementary Material 3. [file 12864_2024_10770_MOESM3_ESM.pdf]

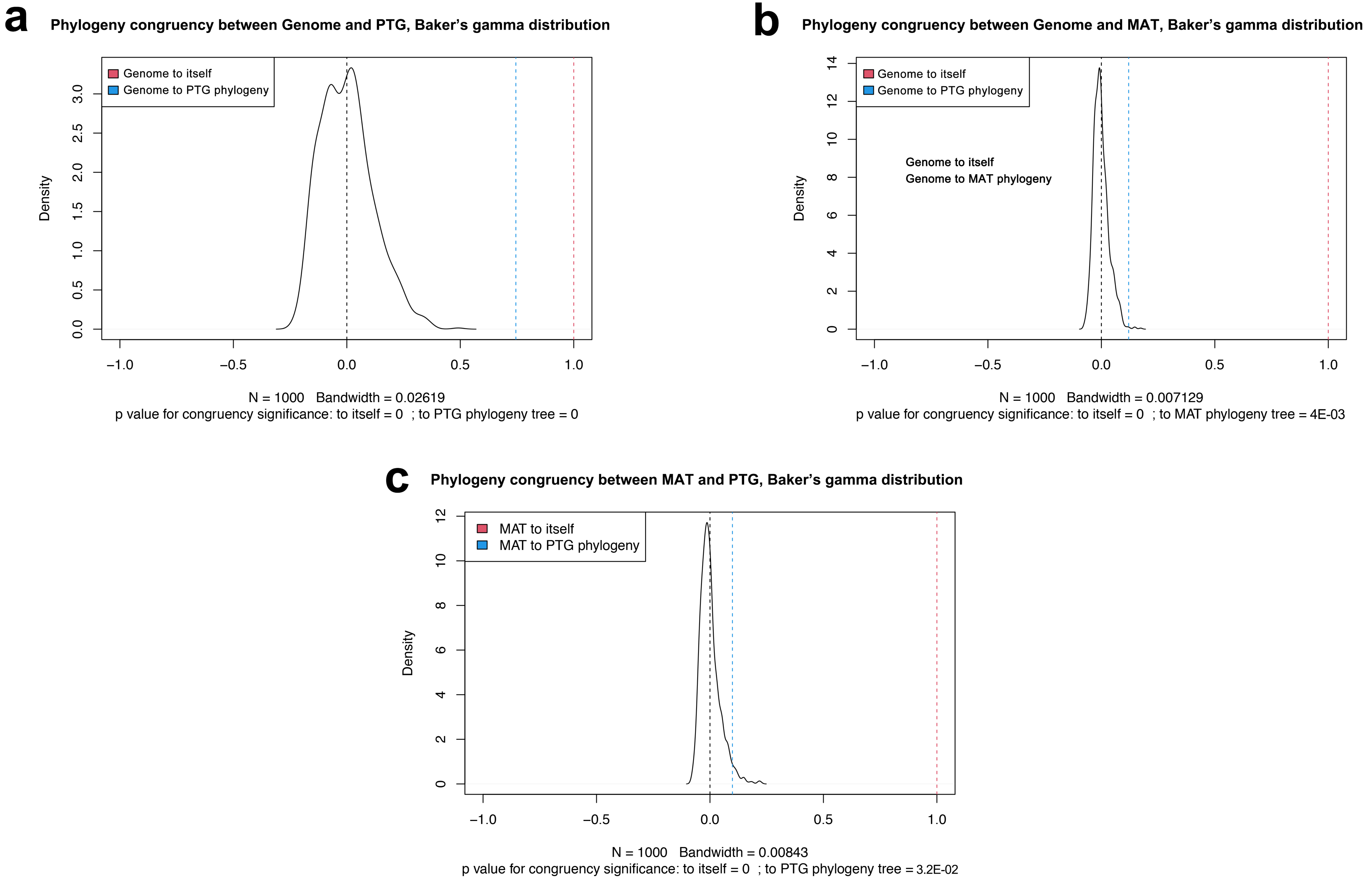

a
Phylogeny congruency between Genome and PTG, Baker's gamma distribution
Genome to itself
Genome to PTG phylogeny
Density
0.0
0.5
1.0
1.5
2.0
2.5
3.0
−1.0
−0.5
0.0
0.5
1.0
N = 1000 Bandwidth = 0.02619
p value for congruency significance: to itself = 0 ; to PTG phylogeny tree = 0
b
Phylogeny congruency between Genome and MAT, Baker's gamma distribution
Genome to itself
Genome to PTG phylogeny
Genome to itself
Genome to MAT phylogeny
Density
0
2
4
6
8
10
12
14
−1.0
−0.5
0.0
0.5
1.0
N = 1000 Bandwidth = 0.007129
p value for congruency significance: to itself = 0 ; to MAT phylogeny tree = 4E-03
c
Phylogeny congruency between MAT and PTG, Baker's gamma distribution
MAT to itself
MAT to PTG phylogeny
Density
0
2
4
6
8
10
12
−1.0
−0.5
0.0
0.5
1.0
N = 1000 Bandwidth = 0.00843
p value for congruency significance: to itself = 0 ; to PTG phylogeny tree = 3.2E-02
